# Does the sex of acute stroke patients influence the effectiveness of rt-PA?

**DOI:** 10.1186/1471-2377-14-60

**Published:** 2014-03-26

**Authors:** Fawaz Al-hussain, Muhammad S Hussain, Carlos Molina, Ken Uchino, Ashfaq Shuaib, Andrew M Demchuk, Andrei V Alexandrov, Maher Saqqur

**Affiliations:** 1 Department of Medicine, King Saud University, Riyadh, Saudi Arabia; 2 Cerebrovascular Center, Cleveland Clinic Foundation, Cleveland, USA; 3 Vall d’Hebron Hospital, Barcelona, Spain; 4 Department of Medicine, University of Alberta, Edmonton, AB, Canada; 5 Department of Clinical Neurosciences, University of Calgary, Calgary, AB, Canada; 6 Department of Neurology, University of Alabama, Birmingham, AL, USA

**Keywords:** Sex, Gender, rt-PA, Thrombolysis, Stroke

## Abstract

**Background:**

Women have been reported to show more frequent recanalization and better recovery after intravenous (IV) recombinant tissue plasminogen activator (rt-PA) treatment for acute stroke compared with men. To investigate this we studied a series of stroke patients receiving IV rt-PA and undergoing acute transcranial doppler (TCD) examination.

**Methods:**

Acute stroke patients received IV rt-PA and had acute TCD examination within 4 hours of symptom onset at 4 major stroke centers. TCD findings were interpreted using the Thrombolysis in Brain Ischemia (TIBI) flow grading system. The recanalization rates, and poor 3-month outcomes (modified Rankin scale >2) of men and women were compared using the chi-square test. Multiple regression analysis was used to assess sex as a predictor of recanalization and poor 3-month outcome after controlling for age, baseline NIH Stroke Scale (NIHSS), time to treatment, hypertension, and blood glucose.

**Results:**

369 patients had TCD examinations before or during IV rt-PA treatment. The 199 (53.9%) men and 170 (46.1%) women had mean ages of 67 ± 13 and 70 ± 14 years, respectively. The sexes did not differ significantly in baseline stroke severity, time to TCD examination, or time to thrombolysis. Of the men, 68 (34.2%) had complete recanalization, 58 (29.1%) had partial recanalization, and 73 (36.6%) had no recanalization. Of the women, 53 (31.2%) had complete recanalization, 46 (27%) had partial recanalization, and 71 (41.8%) had no recanalization (p = 0.6). Multiple regression analyses showed no difference between the sexes in recanalization rate, time to recanalization, or clinical outcome at 3 months.

**Conclusions:**

In our study; sex is not a significant predictor of recanalization rate, time to recanalization or 3-month outcome in stroke patients following IV rt-PA.

**Trial registration:**

Data from CLOTBUST trial Clinicaltrails.gov Identifier: NCT01240356.

## Background

Intravenous (IV) tissue plasminogen activator (rt-PA) remains the only treatment approved in North America for acute ischemic stroke, with a number needed to treat of 8 to reverse 1 stroke completely at 3 months [1]. However, half of rt-PA treated patients remain severely disabled or die within this time. Several factors including stroke severity, older age, systolic hypertension, extent of hypodensity or brain swelling on pretreatment CT, and admission hyperglycemia have been shown to predict poor outcome in stroke patients treated with rt-PA [2-5]. On the other hand, early arterial recanalization is strongly associated with early neurological improvement, reduced infarct size and favorable early and long-term outcome after IV thrombolysis [6-8].

It has been suggested that sex may influence outcome in IV rt-PA treated patients. Large epidemiological studies found that women with stroke have worse functional outcomes compared with men [9-12]. These findings could be explained by sex-based differences in coagulation and fibrinolysis parameters [13]. Controversially; a meta-analysis from the National Institute of Neurological Disorders and Stroke (NINDS), European Cooperative Acute Stroke Study II (ECASS II), and Alteplase Thrombolysis for Acute Non-interventional Therapy in Ischemic Stroke (ATLANTIS) studies suggested that women have more functional improvement 3 months after receiving IV rt-PA within 6 hours from onset of acute stroke compared with men [14], and both sexes, however, have similar clinical outcomes at 3 months. Another study indicated female sex as a predictor of major neurological improvement after IV rt-PA [15]. Major neurological improvement in this study was defined as National Institutes of Health Stroke Scale (NIHSS) score equal to 0 or 1 at 24 hours or an improvement >/= 8 points compared with baseline. One possible explanation could be that females are more likely re-canalize or canalize earlier following IV t-PA. This hypothesis is consistent with Savits SI study that compared recanalization rates after IV t-PA, within 6 hours of stroke onset, using CT/CTA or MRI/MRA to identify major vascular occlusive lesions within the anterior circulation, found that arterial occlusions in women were more likely to recanalize than were those in men [16].

Transcranial Doppler ultrasound (TCD) is a non-invasive test that can be used in the emergency room to evaluate blood flow velocity within the major cerebrovascular arteries. It can quickly determine if intra-arterial occlusion is present or whether recanalization has been achieved using previously validated criteria [17-19].

The aim of our study is to evaluate whether sex differences play a role in predicting immediate and 3-month outcomes in IV rt-PA treated patients. We evaluated differences in the rate and timing of recanalization using CLOTBUST study population and protocol [20]. Our study’s hypothesis is that sex of the patients influence the effectiveness of IV t-PA treatment.

## Methods

### Study approval

The research protocol was approved by the relevant Internal Review Board (IRB) in all four participating hospitals. Appropriate written consent was achieved from candidate patients. Consent was signed by family member where the patient is incompetent due to the acute stroke [20].

### Patients and clinical assessments

The present analysis included all rt-PA treated patients with symptoms of acute cerebral ischemia caused by an intracranial artery [middle cerebral artery (MCA), terminal internal cerebral artery (TICA), anterior cerebral artery (ACA), basilar artery and vertebral artery] with occlusion on pre-treatment TCD according to the criteria validated by our group [18]. Patients with no occlusion on their TCD (e.g. lacunar strokes) or no temporal window for TCD were excluded from our study. Clinical and ultrasound data were prospectively collected at four academic stroke centers (Houston, Barcelona, Edmonton and Calgary) and entered into a multicenter database. Our multicenter database was designed using the CLOTBUST trial methodology [17].

Before IV rt-PA bolus, an experienced sonographer certified by the American Society of Neuroimaging or TCD Flow Grading Examination (at Health Outcomes Institute, 2000) identified residual flow signals at the presumed thrombus location using the Thrombolysis in Brain Ischemia (TIBI) flow grading system. A 2-MHz transducer was positioned at the constant angle of insonation with a standard head frame (Marc series; Spencer Technologies, Seattle, WA). The depth that displayed the worst residual TIBI flow signal was selected. Patients were either continuously monitored with TCD for 2 hours starting before IV rt-PA bolus or underwent intermittent TCD testing every 10–30 minutes using a previously published protocol.

### Transcranial doppler examinations

The follow-up TCD findings were identified as persistent arterial occlusion, partial recanalization, complete recanalization, and re-occlusion. Arterial recanalization on TCD was determined using previously validated criteria [17-19]. In brief, complete recanalization was diagnosed when a normal waveform or a low resistant stenotic signal appeared at the selected depth of insonation (TIBI 4 or 5) suggesting low resistance of the distal circulatory bed. These flow findings correlate with unobstructed passage of contrast agent on angiography. Partial recanalization was diagnosed if the abnormal signals (high resistance dampened signals or flattening of the systolic upstroke with blunted waveform) were still seen at the distal portion (TIBI 2 or 3). No change in the abnormal flow signal indicated that no recanalization had occurred, with minimal flow signal or absent flow corresponding to complete arterial occlusion on angiography (TIBI 0 or 1). These TCD criteria for thrombolysis-associated recanalization in the proximal MCA have been shown to have 91% sensitivity and 93% specificity when compared with angiography [19].

A sonographer first suspected re-occlusion when a decrease in the flow signal by ≥1 TIBI grade was seen on TCD display after complete or partial recanalization and vital signs remained stable. Worsening of flow signal by one TIBI grade indicates an increase in resistance to flow and therefore progression in the degree of arterial obstruction. Systemic reasons for worsening TCD flow (hypotension, bradycardia, low cardiac output, fever) were excluded by closely monitoring the patient’s vital signs, cardiac status and chest X-ray. Clinical outcomes was assessed by blinded study neurologists using modified Rankin Scale (mRS) scores only at 3 months.

### Statistical analysis

Descriptive statistics were expressed as means ± standards deviation (SD) and medians with range for continuous variables, and as numbers (percentages) for categorical variables. Univariate analysis was performed using the two-sample *t* test, chi-square test, and Fisher exact test as appropriate. Multiple logistic regression was used to identify sex as a predictor for outcome after adjusting for confounding factors (age, baseline NIHSS systolic blood pressure (SBP), baseline glucose and onset to IV rt-PA treatment time (OTT)). Age baseline NIHSS, SBP, baseline glucose and onset to treatment time were entered as continuous variables in the multiple logistic regression analysis, whereas TCD flow was entered as a categorical variable. Results were considered significant if the two sided p-value was <0.05. The statistical package SPSS 16 (September 2008 release) was used for data analysis.

## Results

All CLOTBUST subjects (126) were included in our study. The rest of subjects (234) were recruited from University of Alberta Hospital using same selection criteria beyond CLOTBUST trial. A total of 369 patients with acute ischemic stroke, 199 (53.9%) men and 170 (46.1%) women (Table 1). Patients enrolled in clinical trials of ultrasound-enhanced IV rt-PA thrombolysis were included in this analysis [21] as were those treated after 3 hours at similar or lower rt-PA doses, i.e. 0.6 mg/kg (maximum 60 mg) using ethics committee approved protocols. A total of 21 patients were treated between 3 and 4 hours: 14 men (7%) and 7 women (4%) without significant statistical difference between the two groups (p = 0.2).

**Table 1:** Patient’s characteristics based on gender

**Factors**	**Males (n = 199)**	**Females (n = 170)**	**P value**
	**53.9%**	**46.1%**	
Age (years)	67 ± 13	70 ± 14	0.014
Baseline NIHSS (mean ± SD)	16 ± 6	17 ± 5	0.4
NIHSS:			0.2
0-5	6	0 (0%)	
6-10	(3%)	26 (15.3%)	
11-15	29 (14.6%)	42 (24.&%)	
16-20	53 (26.6%)	61 (35.9%)	
>20	64 (32.2%)	41 (24.1%)	
Systolic BP (mean ± SD)	157 ± 21	158 ± 23	0.7
Glucose (mean ± SD)	140 ± 55	159 ± 88	0.012
Glucose >200 mg/dl	19 (10%)	34 (21.5%)	0.004
Diabetes mellitus	33/143 (23.1%)	52/123 (42.3%)	0.001
ASPECT score on CT	8.4 ± 2	8.5 ± 1.4	0.6
Time from stroke onset to IV t-PA (mean ± SD) minutes (median ± SD) minutes	143 ± 73	142 ± 41	0.9
	142 ± 41	143 ± 73	
Time from stroke onset to TCD exam:			0.9
(mean ± SD) minutes	140 ± 84	140 ± 64	
(median ± SD) minutes	136 ± 4	134 ± 96	
Stroke TOAST classification:			0.6
Large artery atherosclerosis	53 (26.6%)	39 (23.1%)	
Cardio-embolism	93 (46.7%)	80 (47.3%)	
Small vessel occlusion	0 (0%)	0 (0%)	
Other: determined	3 (1.5%)	5 (3%)	
Unknown	50 (25.1%)	45 (26.6%)	
Type of occlusion:			0.13
MCA M1	87 (44%)	95 (56%)	
MCA M2	62 (31%)	38 (22%)	
T. ICA	6 (3%)	10 (6%)	
Tandem ICA/MCA	37 (19%)	24 (14%)	
Basilar artery	4 (2%)	1 (0.6%)	
Vertebral artery	2 (1%)	1 (0.6%)	
PCA or ACA	1 (0.5%)	1 (0.6%)	
TCD flow findings:			0.6
Persistent occlusion	73 (36.6%)	71 (41.8%)	
Partial recanalization	58 (29.1%)	46 (27%)	
Compete recanalization	68 (34.2%)	53 (31.2%)	
Time from IV tPA bolus to complete recanalization (mean ± SD) minutes	134 ± 96	136 ± 74	0.8
Clinical deterioration following treatment improvement	26 (13%)	18 (11%)	0.4
Arterial re-occlusion	28 (14%)	29 (17.1%)	0.9
S ICH	17 (8.5%)	13 (7.6%)	0.7
Good long-term outcome (mRS < at 3 months)	78/160 (49%)	65/142 (46%)	0.6

Female subjects were slightly older than males (P = 0.014) correlates the known stroke epidemiology and the fact females get stroke at older age (see Table 1). The mean glucose level and the percentage of patients who presented with a glucose level >200 mg/dl at baseline were higher in the women, which correlates with their higher diabetes prevalence. In both groups, most strokes were secondary to cardio-embolism or large artery atherosclerosis and involved the anterior circulation (Table 1).

**Figure 1:**
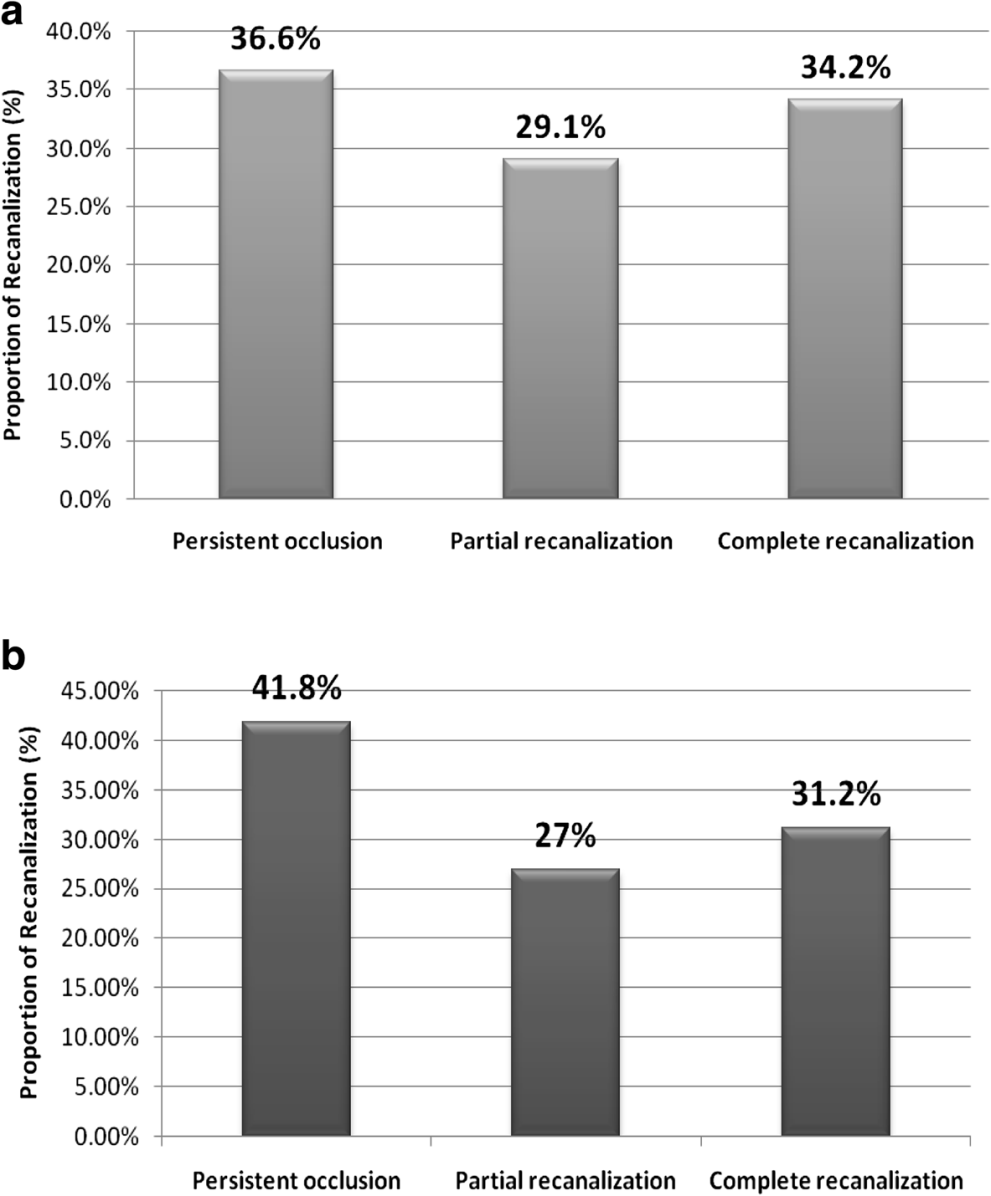
Rate of arterial recanalization after IV rtPA treatment based on gender (a) male (b) female.

TCD findings revealed that in men, 68 of 199 patients (34.2%) had complete recanalization, 58 (29.1%) had partial recanalization, and 73 (36.6%) had persistent occlusion after IV rt-PA treatment. In women, 53 of 170 patients (31.2%) had complete recanalization, 46 (27.0%) had partial recanalization, and 71 (41.8%) had persistent occlusion (p = 0.9) (Figure 1).

Good long-term outcome (mRS ≤2 at 3 months) were seen in 78 of 160 (49%) men and 65 of 142 (46%) women (p = 0.6). In the multiple logistic regression analysis after controlling for common stroke risk factors (age, baseline NIHSS, time to treatment, blood pressure, baseline glucose), sex was not a predictor either of complete recanalization (0.99) or of poor long-term outcome (p = 0.9).

## Discussion

**Figure 2:**
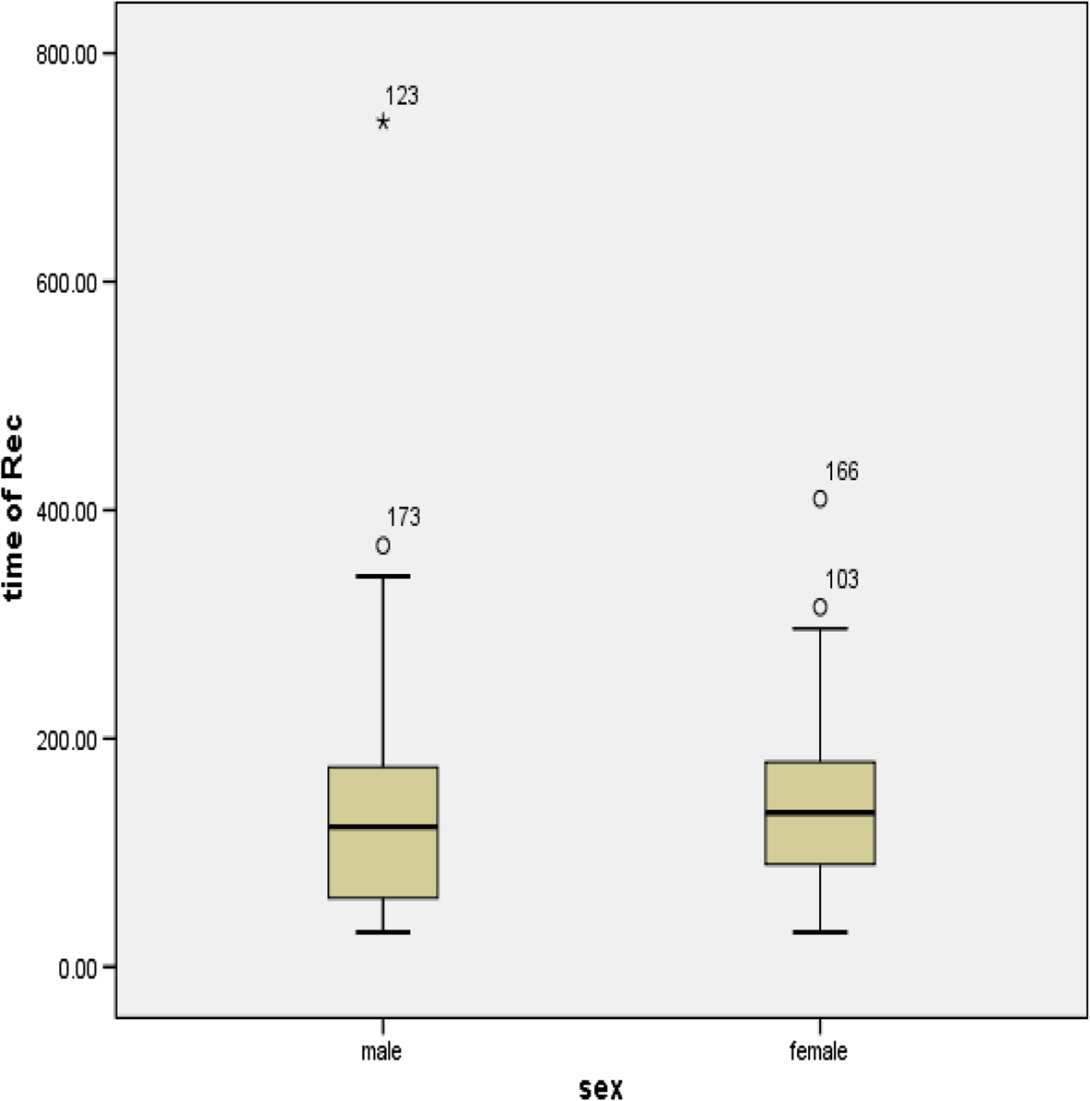
Box whistler plot: median time of arterial recanalization after IV rtPA treatment based on gender.

We found that rates of complete arterial recanalization and good long-term outcome after IV rt-PA were similar in men and women. We also found that there was no sex-based difference in the timing of recanalization (Figure 2), a finding that has not, to our knowledge, previously been published. These results are consistent with those of Shobha et al. [21], who found, in their retrospective analysis of stroke patients from the registry of the Canadian Stroke Network, that sex was not a factor determining outcome after IV thrombolysis.

Our study also showed similar rates for poor clinical outcome (mRS > 2) at 3 months in men and women, consistent with the similar rates of partial and complete recanalization after IV rt-PA in men and women. Our results contrast with those of the previous European study of 4499 patients with first lifetime stroke, which found that 3- month outcomes were poorer and mortality rates higher in women compared with men [12]. The study population may explain the findings in the latter study; women were older, more frequently institutionalized before they presented with stroke, and less likely to have appropriate stroke workup and treatment compared with men. In our study, the rate of poor clinical outcome at 3 months was relatively higher in both sexes when compared with the NINDS trial. This may be because our study population included only patients with proximal intracranial arterial occlusion whereas the NINDS trial included all stroke subtypes, including lacunar stroke.

There are some possible limitations of our study. TCD is an operator-dependent device requiring special training for its application in the acute stroke setting. This was addressed, however, by ensuring that all sonographers were well trained and certified in the application of TCD. Also, in patients with vertebra-basilar artery and ACA occlusions, TCD-monitoring was performed without a head frame, and it may be argued that the monitoring of recanalization in such patients was less reliable compared with patients with MCAs and PCAs where a head frame was applied. Since some patients in our study population received continuous TCD monitoring and others intermittent monitoring, this may have confounded our findings since continuous ultrasound monitoring might augment the thrombolytic efficacy of rt-PA [20]. An unknown number of patients were excluded from this study if they did not have demonstrable occlusion on TCD or adequate TCD quality (inadequate temporal window). The retrospective nature of the analysis precludes any information on patients for whom the TCD operator failed to find a reliable signal. The outcomes of these patients are not known. Follow-up data at 3 months were available for 83% of patients; it is possible that patients for whom follow-up data are not available may have had different outcomes, and this has not been accounted for. The TCD sonographer and investigator were not blinded to the sex of the patients and could have introduced bias into our result. Our analysis is retrospective and thus residual confounding cannot be excluded. We tried to eliminate this possible influence by adjusting for common known confounders in our stepwise logistic regression model. However, our study may be under powered since it is retrospective and a sample size calculation and prior hypothesis were not generated before the study started. We would thus recommend that our findings be confirmed in a future prospective study. Finally; the 3-month clinical outcome measure used was mRS only while using more detailed measures like Barthel Index or NIHSS can show more distinctive results.

## Conclusion

Our findings suggest that sex might not be a predictor of arterial recanalization and clinical outcome in patients with acute ischemic stroke treated with IV rt-PA. Thus sex should not be used as a discriminating factor for treatment or to predict outcome.

## Abbreviations

IV: Intravenous; rt-PA: Recombinant tissue plasminogen activator; TCD: Transcranial doppler; TIBI: Thrombolysis in Brain Ischemia.

## Competing interests

The authors declare that there are no competing interests.

## Authors’ contributions

FAH and MS contributed in writing the proposal and drafted the manuscript. All authors participated in recruiting cases, data collection, and data analysis. MH, CM, KU, AS, AD and AA shared part in writing the manuscript. All authors read and approved the final manuscript.

## Pre-publication history

The pre-publication history for this paper can be accessed here:

http://www.biomedcentral.com/1471-2377/14/60/prepub
